# Pediatric Case of Facial Nerve Palsy as a Complication of Acute Otitis Media Caused by Non-typeable Haemophilus Influenza

**DOI:** 10.7759/cureus.76953

**Published:** 2025-01-05

**Authors:** Milena Mitkova, Julide Kasaboglu, Silvia Valcheva, Raina Gergova, Diana Popova, Alexandra Alexandrova

**Affiliations:** 1 Department of Otolaryngology, Head and Neck Surgery, Medical University-Sofia, Sofia, BGR; 2 Department of Microbiology, Medical University-Sofia, Sofia, BGR

**Keywords:** acute otitis media, facial nerve palsy, haemophilus influenzae, otitis media complication, pediatric

## Abstract

Facial paralysis is an infrequent and serious potential complication of acute otitis media (AOM). We describe a pediatric case of rapidly progressive facial paralysis as a secondary complication alongside AOM, caused by the non-typeable *Haemophilus influenzae* (NTHi) strain, which was managed with facial nerve decompression, glucocorticoid medication, and antimicrobial chemotherapy. The reasons why NTHi becomes pathogenic in certain patients are not yet fully understood, and the specific interactions and adaptations that lead to complications must be further investigated, as they result in more complex treatment approaches.

## Introduction

Acute otitis media (AOM) is a common infectious disease in childhood. AOM is defined by the presence of middle-ear fluid, accompanied by a sudden onset of physical signs and symptoms such as pain, irritability, and fever resulting from inflammation of the middle ear [1,2]. Both bacterial otopathogens and respiratory viruses interact and play an important role in AOM development. While AOM is associated with bacterial etiology in approximately two-thirds of cases, recent studies have increasingly highlighted the significant role of viruses in the development of facial nerve palsy (FNP), with the herpes simplex virus and influenza virus being the most frequently implicated [3]. The most common bacterial causes of AOM are *Streptococcus pneumoniae*,* Haemophilus influenzae*,* Moraxella catarrhalis*,* Staphylococcus aureus*,* Streptococcus pyogenes*,and less frequently,* Pseudomonas species* [1,4].

The pathophysiology of FNP remains unclear. An estimated 15-40 per 100,000 people per year are affected by FNP [5]. Compression of the facial nerve in the facial canal, particularly the narrow labyrinthine segment, is the most widely accepted theory [6]. Other possible reasons are linked to viral infections and potential bacterial complications, autoimmunity, inflammation, and microvascular disease [3,7].

Serious complications of AOM such as acute mastoiditis, facial paralysis, meningitis, and brain abscesses, are rare [1-3]. Their incidence has decreased due to the widespread use of antibiotics and improved disease management. Today, facial paralysis (FP) is an uncommon complication of acute middle ear inflammation, with an estimated incidence of 0.004% to 0.005% [8].

The infrequency of facial nerve palsy makes its treatment a topic of ongoing debate, particularly regarding the necessity of surgical intervention [9].

## Case presentation

A two-year-old child was admitted to the Otorhinolaryngology ward at University Hospital "Queen Joanna"-Sofia due to peripheral paresis of the left facial nerve, which occurred during an episode of AOM.

**Figure 1:**
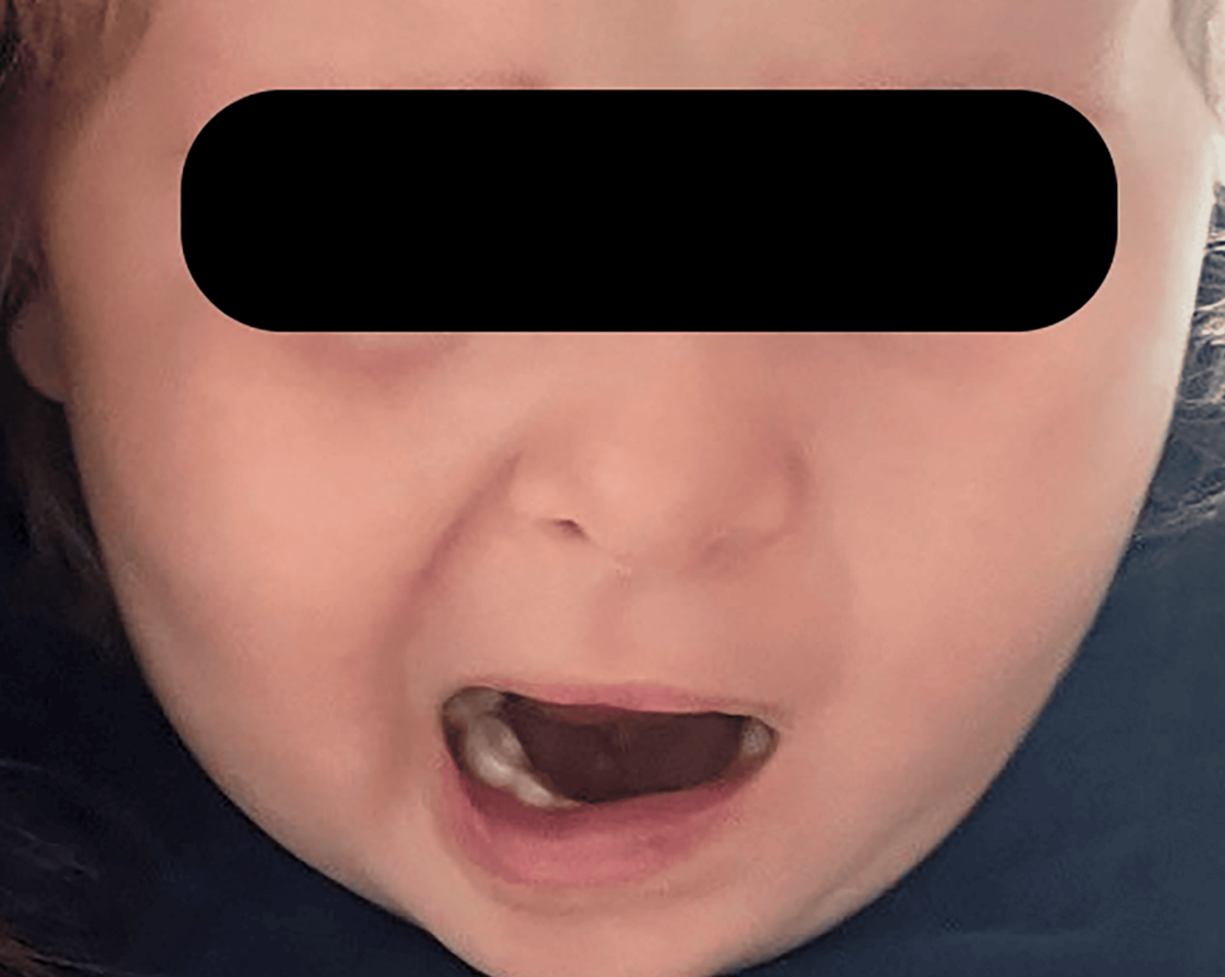
Facial nerve palsy - a complication of acute otitis media caused by NTHi NTHi: non-typeable Haemophilus influenzae

**Figure 2:**
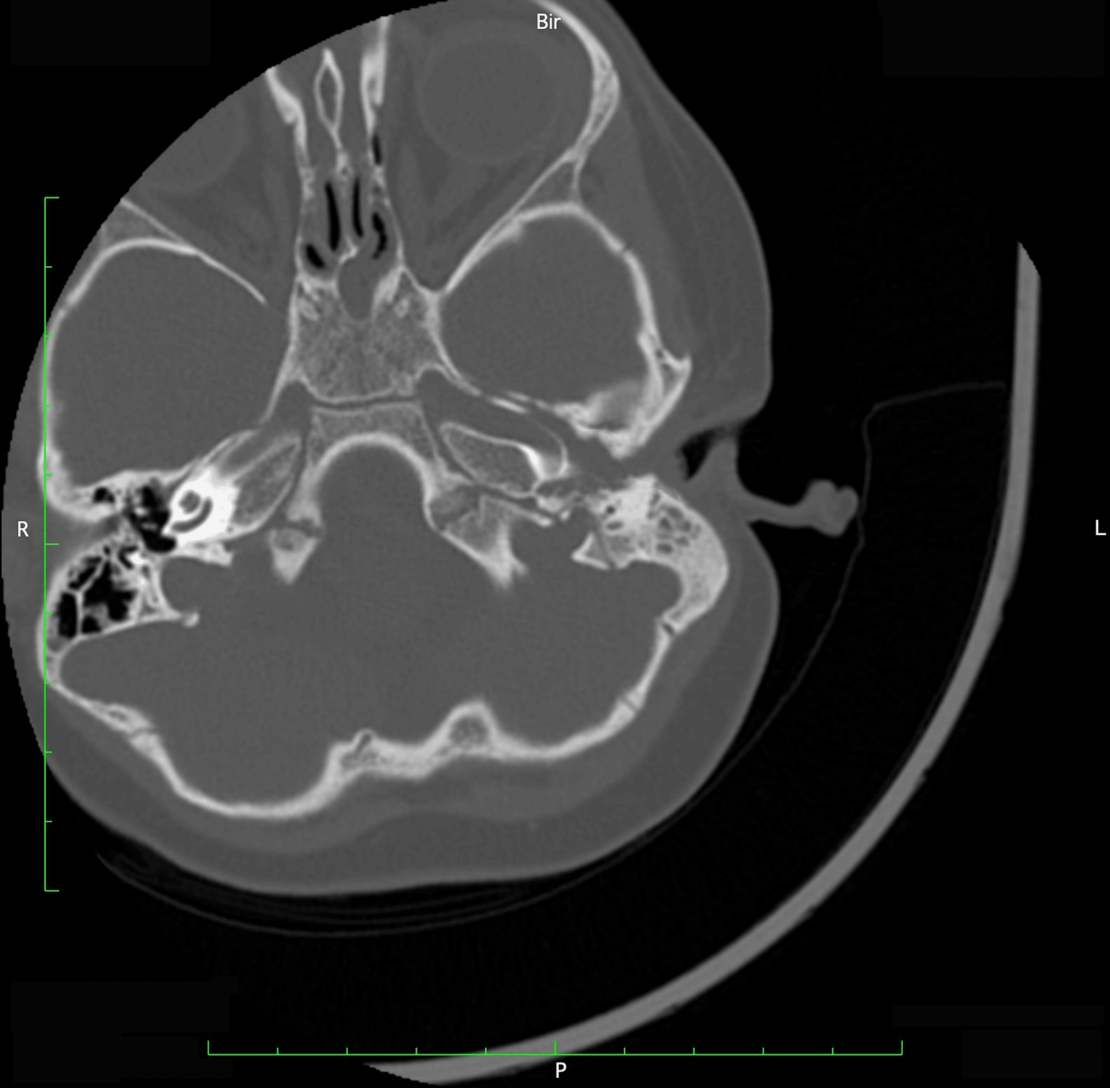
A computed tomography scan of the head Left-sided middle ear and mastoid effusion with complete opacification of the mastoid air cells. Notably, the tympanic cavity showed no erosion of the bony walls in the middle ear or external auditory canal. (L-left; R-right)

Before being admitted to the hospital, the child visited an otorhinolaryngologist because of symptoms including fever, irritability, and pain in the left ear. After the physical examination, treatment with amoxicillin/clavulanate p.o., phenazone, and lidocaine hydrochloride including ear drops, and a nasal decongestant was prescribed. On the sixth day of antibiotic treatment, there were symptoms of false palsy and the child was hospitalized in a Neurology ward. Upon admission, the child was in good general condition and afebrile. Asymmetry in the face was noted, with a slight droop at the left corner of the mouth. The left eyelid did not close during sleep while the forehead muscles remained unaffected. According to the House-Brackmann facial paralysis scale, he was evaluated as Grade IV (Figure 1) A computed tomography (CT) scan of the head was performed, which showed a left-sided middle ear, mastoid effusion, complete opacification of the mastoid air cells, and a tympanic cavity without any erosion of the bony walls in either the middle ear or the external auditory meatus (Figure 2). The child was referred for treatment in an otorhinolaryngology ward. After an otoscopic examination, it was determined that purulent otitis was persistent. Otoscopy revealed an opaque left tympanic membrane, obscuring anatomical details, and a right tympanic membrane, which was also opaque, with slight infiltration in the upper quadrants. There were no vesicles in the ear canal, nor was there swelling in the parotid region.

Additionally, there was no retro auricular edema or erythema. No displacement of the pinna or tenderness in the mastoid area was observed. A bilateral myringotomy was conducted by the otorhinolaryngologist.

Treatment

After the initial complaints and physical examination, a 10-day antibiotic treatment with amoxicillin/clavulanate (250 mg/62.5 mg) was administered at a dosage of 5 ml twice daily. Following the diagnosis of facial nerve palsy, a neurologist prescribed a 15% solution of mannitol and dexamethasone. In the otorhinolaryngology ward, intravenous meropenem was prescribed at a dosage of 40 mg/kg/day, divided into 3 doses for 10 days. This treatment was accompanied by intravenous steroids in the form of methylprednisolone at a dosage of 1 mg/kg/day, administered as scheduled. To protect the left eye from corneal damage, artificial tears were applied several times a day, supplemented with an ophthalmic lubricant ointment and an eyelid patch during the night.

Lab tests and microbiological examination

A complete blood count (Table 1) and microbiological examination were administered.

**Table 1:** Complete blood count (CBC) results obtained on the first day and halfway through the hospital stay MCH (mean corpuscular hemoglobin), MCHC (mean corpuscular hemoglobin concentration), MCV (mean corpuscular volume), MPV (mean platelet volume), PCT (procalcitonin test), PDW (platelet distribution width), RDW (red cell distribution width), Ba (basophils), Eo (eosinophil), RBC (red blood cells), WBC (white blood cells), Lym (lymphocytes), Mo (monocytes), Neu (neutrophils), ESR (erythrocyte sedimentation rate), PLT (platelet count test), HCT (hematocrit test), HGB (hemoglobin), ALAT (alanine aminotransferase test), ASAT (aspartate aminotransferase), CRP (C-reactive protein)

Blood test	1st day	5th day	Reference value
MCH	25	27 pg	26.5 – 32.0
MCHC	328.800	344.500	295 - 360
MCV	77	77	82 - 98
MPV	5.6	5.8	5.9 - 10.0
PCT	0.35	0.35 L/L	0.00 - 0.45 L/L
PDW	17.96	18.37	25 - 65%
RDW	12.5	12.9%	11.5 - 14.5
Ba %	0.95	1.57%	0 - 2%
Ba count	0.11	0.26 G/L	0.00 - 0.20 G/L
Eo %	0.14	0.33%	0 - 6%
Eo count	0.02	0.05 G/l	0.00 - 0.20 G/l
RBC	4.80	4.11 T/l	3.7 - 5.4 T/l
WBC	16.37	12.11 G/l	3.5 - 10.5 G/l
Lym %	18.42	39.22%	20 - 40%
Lym %	2.23	6.42 G/l	0.6 - 4.1 G/l
Mo %	2.25	8.96%	3 - 13%
Mo count	0.27	1.47G/l	0.20 - 1.50 G/l
Neu %	78.25	49.92%	44 - 76%
Neu count	9.47	8.17 G/l	2.0 - 7.8 G/l
ESR	10	10 mm/h	>20 mm/h
PLT	617.3	599.9 G/l	130 - 440 G/l
HCT	0.37	0.32 L/L	0.35 - 0.44 L/L
HGB	121.6	109.7 g/L	115 – 160 g/l
ALAT (GPT)	22	13 U/l	0 - 42
ASAT	31	27 U/l	0 - 41
Creatinine	38	34 umol/L	M 50 - 133; F40 - 120
Urea	6.8	8.5 mmol/l	1.7 - 8.3
CRP	62	47 mg/L	<10 mg/L

The high Neu count and CRP of more than 50 mg/L suggest that a bacterial infection was present. The bacterial cultures of the purulent drainage were positive for *Haemophilus influenzae*. The strain was subjected to detailed microbiological and molecular testing. 

Microbiological Identification

The strain was identified by Remel Rapid NH biochemical identification tests (Thermo Fisher Scientific, Waltham, MA, US). Identification of the capsule-expressing strains was done by polymerase chain reaction detection for the gene encoding the BexB protein, responsible for the intracellular transport of the capsule and, thereby, positive in encapsulated and negative in non-capsulated strains [10].

Antimicrobial susceptibility testing and detection of β-lactamase-encoding genes. The susceptibility testing was performed by an antimicrobial susceptibility plate (Sensititre HPB1 Plate, HTM (T3470), Thermo Fisher Scientific). The results are listed in Table 2. PCR detection of bla-TEM-1 and bla-ROB-1 genes, for the presence of a beta-lactamase, was prepared, as described previously [11].

**Table 2:** In vitro susceptibility testing with a minimal inhibitory concentration test of Haemophilus influenzae isolate recovered from the patient with AOM S-Susceptible; R-Resistant; AOM-acute otitis media *Interpretation was done according to the criteria of EUCAST, 2023 (https://www.eucast.org/clinical_breakpoints).

Antimicrobial agent	MIC mg/L	Interpretation
Ampicillin/Sulbactam 2:1 ratio	1/0.5	S
Ampicillin	2	R
Amoxicillin/Clavulanic Acid 2:1 ratio	2/1	S
Cefuroxime	2	R
Cefaclor	4	R
Ceftriaxone	0.125	S
Cefepime	0.12	S
Cefixime	0.125	S
Chloramphenicol	0.5	S
Clarithromycin	2	R
Erythromycin	0.5	R
Trimethoprim/Sulfamethoxazole	0.5/9.5	S
Levofloxacin	0.03	S
Sparfloxacin	0.03	S
Imipenem	0.5	S
Meropenem	0.12	S
Tetracycline	0.25	S

PCRs for the bexB gene and proof of the absence of a capsule showed the strain was non-typeable Haemophilus ​​​​​influenza (NTHi). The PCR amplifications for the mechanism of resistance revealed beta-lactamase positive NTHi with a TEM - 1 β-lactamase.

Recovery

The patient gradually recovered from paralysis and remained fever-free throughout the hospitalization. The antibiotic treatment with intravenous meropenem and metronidazole lasted a total of 10 days, excluding meropenem on the fifth day, after which the patient was discharged. The parents were instructed to keep the child’s ears dry. Additionally, the child was trained to perform facial muscle exercises and received tactile stimulation under the supervision of a physical therapist.

**Figure 3:**
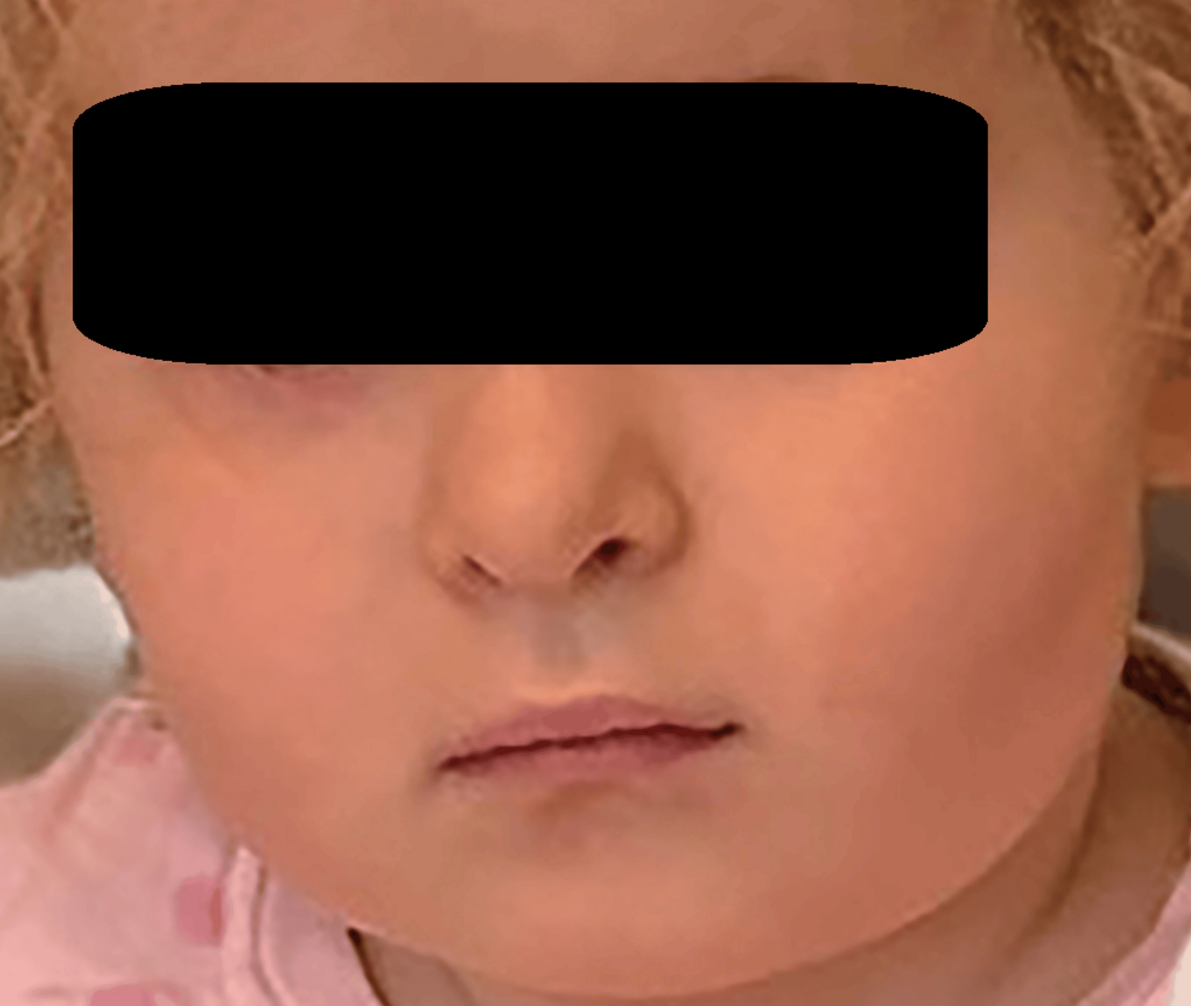
The child was discharged with improvement in facial nerve paresis

Follow-ups were done on the seventh, fourteenth, thirtieth, and ninetieth days after the discharge. During the follow-up examination, by the seventh day after discharge, the tympanic membranes were intact and unfiltered. The child was actively monitored by a pediatric neurologist. After three months, the facial paralysis was completely restored (Figure 3).

## Discussion

This case describes a child's facial nerve palsy as a secondary complication of AOM caused by NTHi.

There is increasing evidence that the predominant causative pathogen in AOM is changing from Streptococcus pneumoniae to non-typeable *Haemophilus influenzae* since the introduction of pneumococcal conjugate vaccines. Additionally, with the introduction of mass immunization against the virulent Haemophilus influenzae type b, which is responsible for serious infections and potentially life-threatening conditions like meningitis, a significant decrease in cases has been observed. Other capsule types, such as a and f, were rarely detected as agents of upper and lower respiratory tract infections [12]. This created an ecological niche that was subsequently filled by NTHi. The proliferation of NTHi has been linked to increased hospitalization rates, greater disease severity, higher morbidity, and notable shifts in the nasopharyngeal microbiome. 

In recent years, NTHi has mainly been identified in recurrent AOM, chronic AOM, as well as cases of complicated antibiotic treatment [1,2]. This case revealed a beta-lactamase positive, ampicillin-resistant strain (BLPAR) with a presence of TEM - 1 β-lactamase, responsible for low-level beta-lactam resistance. NTHi is an adaptable commensal that binds to various host proteins through epithelial attachment and serum factor capture. This enhances its adhesion to host cells, promoting colonization and providing defensive strategies like evading the immune response and forming biofilm-like colonies [13].

The cause of facial nerve palsy in patients with AOM is not fully understood, although several hypotheses have been proposed. First, during the early stages of AOM, the infection may retrogradely spread to the facial nerve canal. Second, inflammatory bacterial toxins may lead to peripheral demyelination of the facial nerve. Lastly, the inflammatory process in the mastoid area can result in inflammation or compression of the facial nerve [13].

Currently, there is no consensus regarding the treatment of facial nerve palsy associated with AOM. The roles of surgical interventions and corticosteroids remain controversial. Some authors advocate for myringotomy, with or without the placement of a tympanostomy tube, in cases of otomastoiditis without tympanic membrane perforation. They also suggest considering mastoidectomy or, in rare cases, decompression of the facial nerve if there is no improvement within a few days.

## Conclusions

Prompt diagnosis and effective treatment are crucial for achieving a good outcome and preventing chronic neurological complications. With this case report, we conclude that the facial nerve palsy associated with acute otitis media (AOM) is generally favorable after appropriate treatment, although about 6% of cases report residual dysfunction. Recovery from facial paralysis typically occurs within three months. Additionally, in cases with an infectious cause or those presenting incomplete symptoms, recovery may happen more quickly.
